# Clinical and Prognostic Values of TRPM7 in Colon and Rectal Cancers

**DOI:** 10.3390/medicina58111582

**Published:** 2022-11-02

**Authors:** Jun-Chae Lee, An-Na Bae, Ha-Jin Lee, Jae-Ho Lee

**Affiliations:** 1 Medical Course, School of Medicine, Keimyung University, Daegu 1095, Korea; 2 Department of Anatomy, School of Medicine, Keimyung University, Daegu 1095, Korea; 3 Chemistry Course, College of Natural Sciences, Keimyung University, Daegu 1095, Korea

**Keywords:** colorectal cancer (CRC), transient receptor potential melastatin 7 (TRPM7), (p53), (APC), (KRAS), The Cancer Genome Atlas (TCGA)

## Abstract

*Background and Objectives*: Transient receptor potential melastatin 7 (TRPM7) is a unique channel protein, and functionally responsible for transportation of calcium and magnesium. Physiologically, the TRPM7 channel is involved in homeostasis of calcium and magnesium, and cell survival. TRPM7 expression is up-regulated in many cancers as malignant behaviors of cancer cells, and its deficiency suppresses their growth. *Materials and Methods*: In this study, we aimed to analyze clinical and prognostic characteristics of TRPM7 expression in colorectal cancers (CRC) using The Cancer Genome Atlas. *Results*: High expression of TRPM7 was observed in younger patients with rectal cancer (*p* = 0.0002). By quantitative correlation analysis, TRPM7 was negatively correlated with age (R = −0.239, *p* = 0.003) and p53 (R = −0.240, *p* = 0.002). Furthermore, it was positively correlated with APC expression (R = 0.534, *p* < 0.001) and KRAS expression (R = 0.319, *p* < 0.001). In colon cancer, there were no variables that showed a significant correlation with TRPM7. Survival analysis found that TRPM7 expression did not have any prognostic value in colon and rectal cancers. *Conclusions*: Our study highlights that TRPM7 expression in CRC, particularly in rectal cancer, may be a potential marker. Future studies are needed to provide deeper insights into the role of TRPM7 in rectal cancer.

## 1. Introduction

Colorectal cancer (CRC) is a common tumor type in South Korea and many developed countries, and is one of the main causes of cancer-related death. Its incidence has increased because of genetic and environmental factors. The adenoma-carcinoma series mainly progresses through adenomatous polyposis coli (APC), Kirsten rat sarcoma virus (KRAS), and p53 mutations [1]. Other types, such as serrated polyps, are associated with microsatellite instability and BRAF mutations [2]. Although the molecular mechanisms of these genetic changes have been studied for a long time, the survival rates of patients with adenocarcinomas have not yet reached a significant level.

Transient receptor potential melastatin 7, as TRPM7, is functionally an ion-channel protein for the transport of calcium and magnesium [3]. Physiologically, the TRPM7 channel is involved in the homeostasis of calcium and magnesium, regulating cell death and survival. TRPM7 expression is upregulated in many cancers as malignant behaviors of cancer cells, and its deficiency suppresses their growth [4]. In previous studies, TRPM7 expression has been found in various cancers, such as glioblastoma, breast, ovarian, nasopharynx, and colon cancers. More big data analysis is needed to elucidate the clinical significance of TRPM7 in various types of cancers. The Cancer Genome Atlas (TCGA) is a research consortium of large-scale genome studies that can be used to investigate various genes [2]. Here, we analyzed clinicopathological and prognostic characteristics of TRPM7 expression and its correlation with the expression of other genes in colon and rectal cancer using TCGA data.

## 2. Materials and Methods

From TCGA website, the microarray, RNA-Seq experiments, and clinical data of colon and rectal cancers were downloaded directly in April 2022. Data of 440 colon cancers and 158 rectal cancers were analyzed. TNM stage was evaluated according to the seventh edition of the American Joint Committee on Cancer staging system.

Statistical analysis was performed using the SPSS software (version 25.0; IBM SPSS, Armonk, NY, USA). Clinicopathological characteristics such as age, carcinoembryonic antigen (CEA) level, sex, lymphatic invasion, venous invasion, and pathologic TNM stage were analyzed using the Chi-square test. The correlation analysis between genes and variables was performed by Spearman’s correlation coefficient analysis. Univariate survival analysis was performed using the log-rank test with Kaplan–Meier curves. Overall survival was defined as the time between diagnosis and mortality. *p* < 0.05 was considered statistically significant.

## 3. Results

To determine the clinical characteristics of TRPM7 expression, patients were divided into two subgroups according to its median value. The characteristics of TRPM7 expression in rectal cancer and the colon are presented in Table 1. Notably, higher TRPM7 expression was observed in younger patients with rectal cancer (*p* < 0.001). It was also associated with lymphatic invasion and T stage; however, the association was not statistically significant. In colon cancer, TRPM7 expression correlated with T stage; however, it was not statistically significant (*p* = 0.072). 

Quantitative correlation analysis between clinical parameters and gene expression was performed. TRPM7 showed a negative correlation to age (R = −0.239, *p* = 0.003) and p53 (R = −0.240, *p* = 0.002) in rectal cancer (Table 2). 

It was positively correlated with APC (R = 0.534, *p* < 0.001) and KRAS (R = 0.319, *p* < 0.001) expression. No variables have a significant correlation to TRPM7 expression in colon cancer (Table 3).

**Figure 1:**
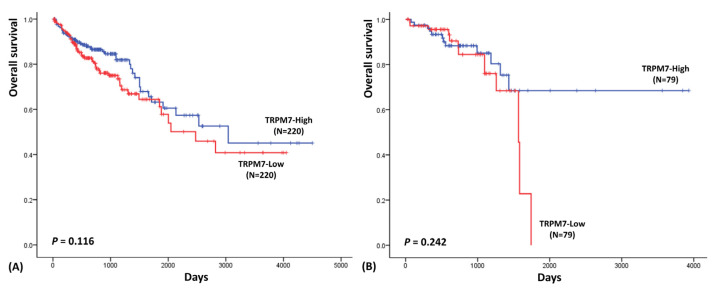
Overall survival analysis of TRPM7 in colon cancer (**A**) and rectal cancer (**B**).

Survival analysis was performed to expose the prognostic value of TRPM7 in colorectal cancer. In overall survival analysis in patients with colon cancer, the expression and significance of TRPM7 levels were not found (χ^2^ = 2.467, *p* = 0.116). Similarly, TRPM7 did not show prognostic significance for rectal cancer (χ^2^ = 1.367, *p* = 0.242). When stratified by other variables, there were no statistically significant differences (Figure 1).

## 4. Discussion

In present study, we demonstrated the clinical characteristics of TRPM7 in colon and rectal cancers using public data (TCGA) for the first time. Ion channels influence the process of cancer cells. Calcium (Ca2+) and magnesium (Mg2+) are important metal elements [5]. Ion channels affect cancer cell biology such as progression and death. Calcium and magnesium are important regulators of cancer development [6]. According to previous research, transient receptor potential (TRP) channels are mainly involved in cationic signaling and cause membrane depolarization, involving numerous physiological functions. Therefore, TRP may be important for carcinogenesis and cancer apoptosis [7]. In particular, TRPM7, a member of the TRP family, is important in cancer cell biology because it mediates calcium and magnesium flows [4,6]. TRPM7 is composed of six transmembrane domains, a pore-forming domain between the fifth and sixth transmembrane domains, a coiled-coil domain involved in the tetramerization of channels, and a COOH-terminal α-kinase domain [6,7]. This channel is involved in the homeostasis of calcium, and magnesium homeostasis [8]. It can phosphorylate numerous target proteins related to cell development, proliferation and migration [9,10]. 

Recent studies have shown that TRPM7 is expressed more highly in cancer tissues than non-cancerous tissue, and its pattern is associated with clinical characteristics in many cancers. Overexpression of TRPM7 has a poorer survival result in breast cancer [4] and has a negative effect on the progressive tumor behavior of gastric cancer and the prognosis of patients [11]. In prostate cancer, high expression of TRPM7 is associated with the migration and invasion of cancer cells [12]. In pancreatic cancer, TRPM7 is highly expressed, correlating to tumor size and stage [13]. TRPM7 was overexpressed in human inflammatory bowel disease-related and sporadic CRC, and it had positive correlation to tumor grade [14]. In CRC, TRPM7 was overexpressed and decreased TRPM7 in vitro [15].

Based on these data, we analyzed the clinical characteristics of TRPM7 expression in colon and rectal cancer. Our data showed that TRPM7 expression was higher in younger patients than that in older patients with rectal cancer. This result is in agreement with the quantitative correlation analysis. The results showed a negative correlation between age and TRPM7 level. Although the difference was statistically significant, the T stage was positively associated with TRPM7 expression. However, survival analysis showed no prognostic value for TRPM7 in colon and rectal cancers. Our study and previous studies have suggested that TRPM7 expression may be associated with the progressive characteristics of CRC [14,15]. The biological changes in TRPM7 should be further studied in larger cases of CRC. 

Interestingly, TRPM7 expression was positively correlated with APC and KRAS gene expression in rectal cancer. However, it is inversely related to p53 expression. There are multiple oncogenic pathways involved in colorectal carcinogenesis; therefore, understanding its molecular etiology is important. It is well known that the expression of APC, KRAS, and p53 is frequently impaired in human cancers, and p53 mutations are found in more than 40% of tumors, especially CRC [13,16]. TRPM7 is closely related to the main genes involved in colorectal carcinogenesis, suggesting that it plays an important role in rectal cancer. To identify the regulation and function of TRPM7 in CRC, further study should be performed on colorectal cancer subtypes.

## 5. Conclusions

In conclusion, our study highlights TRPM7 expression in CRC, particularly in rectal cancer, as a potential genetic marker. This study suggest TRPM7 has a critical role in the pathogenesis of rectal cancer, and may become a potential biomarker and therapeutic target for cancer.

## Figures and Tables

**Table 1:** Clinical characteristics of TRPM7 in colon and rectal cancers.

	TRPM7 (Rectal Cancer)			TRPM7 (Colon Cancer)		
	High (N,%)	Low (N,%)	*p*-Value	High (N,%)	Low (N,%)	*p*-Value
Age						
<60	35 (71.4%)	14 (28.6%)	<0.001	89 (52.4%)	81 (47.6%)	0.433
≥60	43 (39.8%)	65 (60.1%)		131 (48.5%)	139 (51.5%)	
Gender						
Female	36 (50.7%)	35 (49.3%)	0.816	103 (50%)	103 (50%)	1.000
Male	42 (48.8%)	44 (51.2%)		117 (50%)	117 (50%)	
Lymphatic invasion						
No	45 (54.9%)	37 (45.1%)	0.092	118 (48.4%)	126 (51.6%)	0.751
Yes	23 (41.7%)	34 (60.7%)		76 (50%)	76 (50%)	
CEA (ng/mL)						
≤5	31 (50.8%)	30 (49.2%)	0.934	98 (51.9%)	91 (48.1%)	0.372
>5	22 (50%)	22 (50%)		42 (46.2%)	49 (53.8%)	
Venous invasion						
No	52 (51%)	50 (49%)	0.407	142 (48.8%)	149 (51.2%)	0.524
Yes	15 (42.9%)	20 (57.1%)		40 (44.4%)	50 (55.6%)	
Pathologic stage						
stage I	10 (33.3%)	20 (66.7%)	0.260	42 (59.2%)	29 (40.8%)	0.384
stage II	23 (50%)	23 (50%)		83 (48.5%)	88 (51.5%)	
stage III	27 (56.3%)	21 (43.7%)		59 (46.8%)	67 (53.2%)	
stage IV	11 (45.8%)	13 (54.2%)		31 (50.8%)	30 (49.2%)	
M stage						
M0	56 (47.5%)	62 (52.5%)	0.464	159 (48.9%)	166 (51.1%)	0.785
M1	9 (39.1%)	14 (60.9%)		31 (50.8%)	30 (49.2%)	
N stage						
N0	35 (44.3%)	44 (55.7%)	0.342	131 (50.8%)	127 (49.2%)	0.283
N1	22 (51.2%)	21 (48.8%)		56 (53.8%)	48 (46.2%)	
N2	19 (59.4%)	13 (40.6%)		33 (42.3%)	45 (57.7%)	
T stage						
T1	1 (11.1%)	8 (88.9%)	0.093	4 (36.4%)	7 (63.6%)	0.072
T2	13 (46.4%)	15 (53.6%)		47 (63.5%)	27 (36.5%)	
T3	55 (51.9%)	51 (48.1%)		144 (47.5%)	159 (52.5%)	
T4	8 (61.5%)	5 (38.5%)		25 (49%)	26 (51%)	

**Table 2:** Correlation analysis in rectal cancer.

		TRPM7	APC	KRAS	p53	Age	CEA
TRPM7	R	1	0.534	0.319	−0.240	−0.239	−0.026
	*p*		<0.001	<0.001	0.002	0.003	0.795
APC	R	0.534	1	0.261	−0.148	−0.222	−0.013
	*p*	<0.001		0.001	0.064	0.005	0.893
KRAS	R	0.319	0.261	1	−0.085	0.014	−0.007
	*p*	<0.001	0.001		0.290	0.857	0.942
p53	R	−0.240	−0.148	−0.085	1	0.078	−0.104
	*p*	0.002	0.064	0.290		0.330	0.290
Age	R	−0.239	−0.222	0.014	0.078	1	0.008
	*p*	0.003	0.005	0.857	0.330		0.932
CEA	R	−0.026	−0.013	−0.007	−0.104	0.008	1
	*p*	0.795	0.893	0.942	0.290	0.932	

**Table 3:** Correlation analysis in colon cancer.

		TRPM7	APC	KRAS	p53	Age	CEA
TRPM7	R	1	0.058	−0.034	0.043	−0.01	0.067
	*p*		0.224	0.554	0.364	0.832	0.265
APC	R	0.058	1	−0.031	0.017	0.002	0.009
	*p*	0.224		0.593	0.727	0.965	0.882
KRAS	R	−0.034	−0.031	1	−0.111	−0.018	−0.115
	*p*	0.554	0.593		0.052	0.748	0.096
p53	R	0.043	0.017	−0.111	1	0.052	0.016
	*p*	0.364	0.727	0.052		0.281	0.793
Age	R	−0.01	0.002	−0.018	0.052	1	0.003
	*p*	0.832	0.965	0.748	0.281		0.965
CEA	R	0.067	0.009	−0.115	0.016	0.003	1
	*p*	0.265	0.882	0.096	0.793	0.965	

## Data Availability

The data presented in this study are available on request from the corresponding author.
